# Cell‐free DNA blood‐based test compared to fecal immunochemical test for colorectal cancer screening

**DOI:** 10.1002/cac2.70037

**Published:** 2025-05-26

**Authors:** Teresa Seum, Michael Hoffmeister, Hermann Brenner

**Affiliations:** ^1^ Division of Clinical Epidemiology and Aging Research German Cancer Research Center (DKFZ) Heidelberg Germany; ^2^ Heidelberg Medical Faculty Heidelberg University Heidelberg Germany; ^3^ German Cancer Consortium (DKTK) German Cancer Research Center (DKFZ) Heidelberg Germany

**List of abbreviations**
APCL: advanced precancerous lesion
cfDNA: cell‐free DNA
CRC: colorectal cancer
FIT: fecal immunochemical test

1

Screening for colorectal cancer (CRC) is among the most effective approaches to cancer prevention, yet achieving high adherence to effective screening offers is challenging [1]. Blood‐based tests that could be easily implemented in routine medical practice might be a promising approach to achieve higher adherence rates than with conventional stool‐based or endoscopic screening [2, 3]. However, neoplasm detection rates of previously developed and proposed blood‐based tests have not been competitive to those of modern stool‐based tests [2], in particular fecal immunochemical tests (FITs) that are meanwhile widely used for CRC screening in an increasing number of countries [4]. Most recently, performance of a novel cell‐free DNA (cfDNA) blood‐based test for detecting colorectal neoplasms was validated in the ECLIPSE study, a large screening population undergoing screening colonoscopy [5], being the first of its kind to achieve FDA approval as a primary screening option for CRC.

Although detailed results on sensitivity and specificity were reported, the ECLIPSE study [5] did not include comparative results for screening by FIT, the best established and most widely used noninvasive CRC screening test. We aimed to compare reported measures of diagnostic performance of the cfDNA blood‐based test to those of a commercially available FIT (FOB Gold, Sentinel Diagnostics, cutoff 17.0 µg hemoglobin per gram feces) in the BLITZ study, a comparable large cohort of screening colonoscopy participants recruited in the context of the German screening colonoscopy program. The complete description of the methods can be found in the Supplementary Materials and Methods.

In order to match the inclusion and exclusion criteria of the ECLIPSE study as closely as possible, analogous exclusion criteria were applied to the BLITZ sample, resulting in 5,683 participants from the BLITZ study to be included in the analysis (Supplementary Figure S1).

Supplementary Table S1 summarizes the characteristics of the ECLIPSE study, conducted in the United States with 7,861 screening colonoscopy participants, and the selected study sample from the BLITZ study. Mean age and sex distributions of participants in the ECLIPSE study (60.3 years, 53.7% women) and the BLITZ study (61.2 years, 50.4% women) were similar, as were the proportions of participants in whom CRC was detected (0.8% in both studies). Advanced precancerous lesions (APCLs) were somewhat more commonly found in the ECLIPSE study (14.2% vs. 10.3%) (Supplementary Table S1).

In the ECLIPSE study, 11.4% of participants were tested positive by the cfDNA blood test, while 9.9% tested positive by the FIT in the BLITZ study (Table 1). Applying the FIT manufacturer's recommended cutoff, the sensitivity of FIT was much higher than the cfDNA test in detecting APCL (31.5% vs. 13.2%, *P* < 0.001) and showed an approximately one third lower false positive rate (specificity 93.3% vs. 89.6%, *P* < 0.001). The FIT also demonstrated higher sensitivity than the cfDNA test for combined CRC and APCL detection (35.4% vs. 17.0%, *P* < 0.001), largely reflecting its performance in detecting APCL. Although the FIT showed a higher sensitivity for overall CRC detection compared to cfDNA (88.6% vs. 83.1%), this difference did not reach statistical significance (*P* = 0.597). Sensitivity for stage I‐III CRC was comparable between the two tests (86.5% for FIT vs. 87.5% for cfDNA, *P* = 1.000).

**TABLE 1:** Diagnostic performance in the ECLIPSE and the BLITZ study.

		Study		
Diagnostic performance		ECLIPSE study^a^, cfDNA blood‐based test (Shield)	BLITZ study, FIT (FOB Gold)	*P* value
Positivity rate, % (95% CI)		11.4 (10.7‐12.1)	9.9 (9.1‐10.7)	0.005
Sensitivity, % (95% CI)	CRC any stage	83.1 (72.2‐90.3)	88.6 (75.4‐96.2)	0.597
	CRC stages I‐III	87.5 (75.3‐94.1)	86.5 (71.2‐95.5)	1.000
	APCL^b^	13.2 (11.3‐15.3)	31.5 (27.7‐35.4)	<0.001
	CRC or APCL^b^	17.0 (14.9‐19.3)	35.4 (31.7‐39.3)	<0.001
Specificity, % (95% CI)	No CRC or APCL^b^	89.6 (88.8‐90.3)	93.3 (92.6‐94.0)	<0.001

Abbreviations: APCL, advanced precancerous lesions; cfDNA, cell‐free DNA; CI, confidence interval; CRC, colorectal cancer; FIT, fecal immunochemical test

^a^ Data extracted from Chung et al. [5].

^b^ Defined as adenomas ≥ 1cm, tubulovillous, or villous adenomas, serrated polyps ≥ 1cm or adenomas with high‐grade dysplasia.

This study compares the performance of a recently developed and validated cfDNA blood‐based test and a long‐established FIT in detecting CRC and its precursors in a CRC screening population. Utmost care was given to maximize comparability by employing closely matching inclusion and exclusion criteria. Our results demonstrate that the cfDNA blood‐based test may come close to the sensitivity of the FIT to detect CRC but is far from being competitive with the FIT in detecting advanced preneoplastic lesions. The specificity of the FIT was also higher than the specificity reported for the cfDNA blood‐based test.

Sensitivity for detection of APCL is a key determinant of a CRC screening test's effectiveness, and is a major challenge for any blood‐based screening tests [3]. The disadvantage in sensitivity for APCL of the cfDNA blood‐based or other blood‐based tests is likely to equally apply in comparison to other established or recently proposed fecal tests, such as the multitarget stool DNA [6, 7] and RNA tests [8, 9]. A potential advantage of blood‐based tests could be easier implementation and higher adherence in routine medical practice. However, the apparent disadvantage in adherence of stool‐based tests such as the FIT may be effectively overcome at comparatively much lower costs by well‐organized screening programs, as meanwhile convincingly demonstrated in multiple countries, such as the Netherlands or Denmark [4]. A recent modeling study showed FIT‐based screening as the more effective and cost‐effective option compared to blood‐based tests even with lower screening uptake [10]. Taken together, for the time being, efforts to achieve high adherence in well‐organized FIT‐based programs appear the more promising approach to enhance the impact of noninvasive CRC screening on the population level. However, further research should aim to enhance diagnostic performance of both blood‐ and stool‐based tests, in particular with respect to the detection of APCLs.

Strengths of our study include its reliance on a large screening cohort in which colonoscopy was conducted in all participants, not just those with a positive FIT value. The main limitation of our study is its reliance on an indirect comparison of both tests in two different study populations. Results could therefore have been affected by differences in study populations and differences in quality of screening colonoscopy. This limitation could have been avoided if the ECLIPSE study would have employed a FIT along with the blood testing. However, although differences in screening colonoscopy quality might have affected detection rates of nonadvanced neoplasms, potential impact on detection rates, sensitivity and specificity of APCL or CRC would be expected to be small.

In conclusion, further major improvement in diagnostic performance is needed for the cfDNA blood‐based test to become a competitive alternative for noninvasive CRC screening. Major efforts to enhance sensitivity of this and alternative “liquid biopsy” approaches to detect preneoplastic lesions would be of paramount importance for efficient use in CRC screening practice.

## AUTHOR CONTRIBUTIONS

*Study design*: Hermann Brenner. *Statistical analyses*: Teresa Seum. *Manuscript writing*: Hermann Brenner and Teresa Seum. *Critical revision of the manuscript for important intellectual content*: Michael Hoffmeister. All authors read and approved the final manuscript.

## CONFLICT OF INTEREST STATEMENT

Hermann Brenner reported receiving grants from the German Research Foundation (DFG), German Federal Ministry of Education and Research (BMBF), and German Cancer Aid during the conduct of the study. No other disclosures were reported.

## FUNDING INFORMATION

This study was partly funded by grants from the German Research Council (DFG, grant No. BR1704/16‐1), the Federal Ministry of Education and Research (BMBF, grant no. 01GL1712 and 01KD2104A), and the German Cancer Aid (No. 70113330). The funding source had no role in the design and conduct of the study; collection, management, analysis, and interpretation of the data; preparation, review, or approval of the manuscript; and decision to submit the manuscript for publication.

## ETHICS APPROVAL AND CONSENT TO PARTICIPATE

The BLITZ study was approved by the ethics committees of the Heidelberg Medical Faculty of Heidelberg University (178/2005) and of the responsible state medical chambers. The study is registered in the German Clinical Trials Register (DRKS‐ID: DRKS00008737). Written informed consent was obtained from each participant.

## Supporting information



Supporting Information

## Data Availability

Individual data cannot be shared due to confidentiality reasons.
