# Supplementary material for: Cell‐free DNA blood‐based test compared to fecal immunochemical test for colorectal cancer screening
Source: Cancer Commun (Lond). 2025 May 26;45(8):987–9. doi: 10.1002/cac2.70037 (PMC12365541; doi:10.1002/cac2.70037)
Supplement: Supplementary file 1 — Supporting Information [file CAC2-45-987-s001.docx]

**Supplementary Materials**

**Cell-free DNA blood-based test compared to fecal immunochemical test**

**for colorectal cancer screening**

Teresa Seum^1,2^, Michael Hoffmeister^1^, Hermann Brenner^1,3,*^

^1^Division of Clinical Epidemiology and Aging Research, German Cancer Research Center (DKFZ), Heidelberg, Germany.

^2^Heidelberg Medical Faculty, Heidelberg University, Heidelberg, Germany.

^3^German Cancer Consortium (DKTK), German Cancer Research Center (DKFZ), Heidelberg, Germany.

^*^**Corresponding author:**

Hermann Brenner; Division of Clinical Epidemiology and Aging Research, German Cancer Research Center.

**Supplementary Materials and Methods**

***Study design and study population***

Our analyses are based on data from the ongoing German BLITZ study whose design and data collection have been reported in detail elsewhere [1, 2]. In the BLITZ study, participants in the German screening colonoscopy program have been recruited by up to 20 gastroenterology practices in Southern Germany since the end of 2005. In Germany, screening colonoscopy has been offered as a primary screening exam for men and women aged 55 or older since 2002 (in 2019 the starting age was lowered to 50 years for men). Up to two screening colonoscopies 10 or more years apart are offered and conducted mostly in gastroenterology practices. Only experienced endoscopists are entitled to do screening colonoscopies which are subject to rigorous measures of quality assurance [3, 4].

For this analysis, we included participants who were recruited in 2008 to 2020 when the same quantitative fecal immunochemical test (FIT; FOB Gold, Sentinel Diagnostics) was applied. Upon written informed consent, participants completed a short questionnaire, and provided a fecal sample prior to bowel preparation for the evaluation of established and novel noninvasive CRC screening tests. The BLITZ study was approved by the ethics committees of the Heidelberg Medical Faculty of Heidelberg University (178/2005) and the state medical chambers of Baden-Württemberg (M118-05-f), Saarland (217/13), Rhineland Palatinate (837.047.06[5145]) and Hesse (MC 254/2007).

In order to match the inclusion and exclusion criteria of the ECLIPSE study as closely as possible, we employed the following analogous exclusion criteria: age < 45 or > 84 years, history of cancer, a known diagnosis of inflammatory bowel disease, a history of CRC in a first-degree relative, colonoscopy within the preceding 9 years, invalid, incomplete or poor colonoscopy, and invalid FIT results.

***Data collection***

Participants completed a standardized questionnaire before colonoscopy, providing information on sociodemographic and lifestyle factors. Colonoscopy and pathology data were independently extracted by two trained research assistants blinded to questionnaire and FIT results. Findings were categorized by the most advanced lesion: CRC, advanced adenoma, sessile serrated polyp ≥ 1 cm, non-advanced adenoma, other, or no finding. Advanced adenomas were defined as adenomas with at least one of the following: size ≥ 1 cm, tubulovillous or villous components, or high-grade dysplasia. Participants with advanced adenomas or sessile serrated polyps ≥ 1 cm were classified as having advanced precancerous lesions (APCL).

Fecal samples were collected before bowel preparation without dietary or medication restrictions. Between 2008 and 2012, participants stored stool samples in containers at home (frozen or refrigerated) and brought them to colonoscopy appointments, where they were frozen at -20°C before shipping on dry ice to a central laboratory. From 2012 onwards, stool samples were directly collected in FIT devices with hemoglobin-stabilizing buffer and mailed to the study center before cold-chain transport to the central laboratory. The median time between stool collection and analysis was 7 days (IQR: 4-10 days). All FIT analyses were performed at the Limbach Laboratory (Heidelberg, Germany) on the Abbott Architect c8000 by experienced personnel blinded to colonoscopy findings. Both collection methods yielded comparable FIT results [5].

***Statistical analysis***

We compared main characteristics of the study participants of the ECLIPSE study and the BLITZ study by descriptive statistics. Sensitivities for CRC, CRC stage I to III, any advanced neoplasia (including CRC and APCL) and APCL as well as specificity for absence of CRC and APCL were extracted from the ECLIPSE study report and derived for the FIT in the BLITZ study using the cut-off recommended by the manufacturer (17.0 µg hemoglobin per gram feces). Differences in performance characteristics of the tests in the two studies were tested for statistical significance by chi-square tests or, in case of small numbers, Fisher’s exact test. All analyses were conducted using R version 4.3.2 (<https://www.r-project.org/>).

**Supplementary Table S1. Study population characteristics of the ECLIPSE and the BLITZ study.**

| **Characteristics** | | **Study** | |
| --- | --- | --- | --- |
|  |  | **ECLIPSE study^a^,**  **cfDNA blood-based test** (Shield) | **BLITZ study,**  **FIT** (FOB Gold) |
| Case No. | | 7,861 | 5,683 |
| Country | | USA | Germany |
| Recruitment, years | | 2019-2022 | 2008-2020 |
| Sex, *n* (%) | Male | 3,643 (46.3) | 2,821 (49.6) |
|  | Female | 4,218 (53.7) | 2,862 (50.4) |
| Age | Range, years | 45-84 | 45-84 |
|  | Mean (SD), years | 60.3 (9.1) | 61.2 (6.8) |
|  | 45-59 years, *n* (%) | 3,695 (47.0) | 2,909 (51.2) |
|  | 60-84 years, *n* (%) | 4,166 (53.0) | 2,774 (48.8) |
| Most advanced finding at colonoscopy, *n* (%) | CRC any stage | 65 (0.8) | 44 (0.8) |
|  | CRC stages I-III | 48 (0.6) | 37 (0.7) |
|  | APCL^b^ | 1,116 (14.2) | 588 (10.3) |
|  | CRC or APCL^b^ | 1,181 (15.0) | 632 (11.1) |
|  | No advanced neoplasia | 6,680 (85.0) | 5,051 (88.9) |

^a^Data extracted from Chung et al. [6].

^b^Defined as adenomas ≥ 1cm, tubulovillous, or villous adenomas, serrated polyps ≥ 1cm or adenomas with high-grade dysplasia.

Abbreviations: APCL, advanced precancerous lesions; cfDNA, cell-free DNA; CRC, colorectal cancer; FIT, fecal immunochemical test; SD, standard deviation.


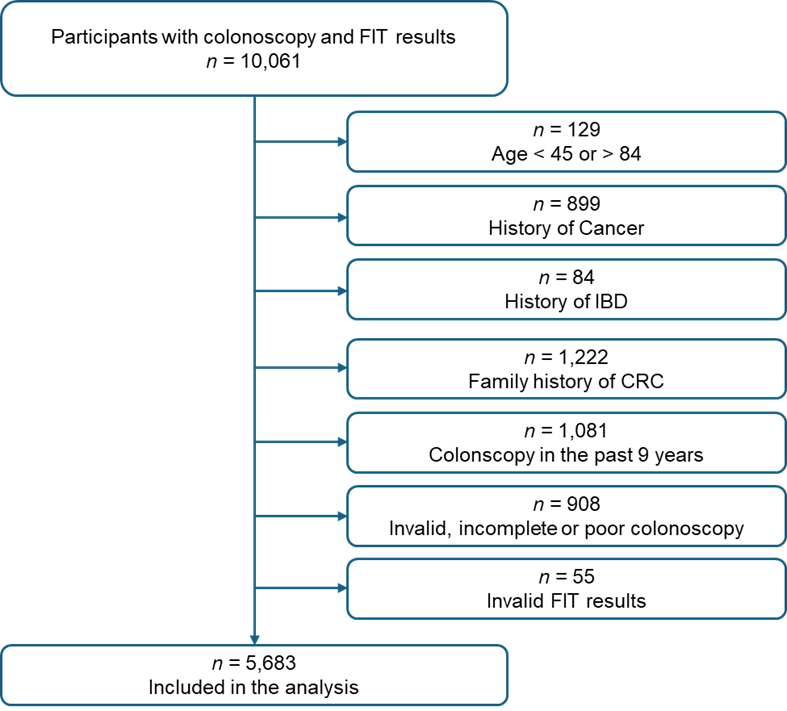


**Supplementary Figure S1.** **Flow diagram.**

Abbreviations: CRC, colorectal cancer; FIT, fecal immunochemical test.

**Supplementary References**

1. Gies A, Cuk K, Schrotz-King P, Brenner H. Direct Comparison of Diagnostic Performance of 9 Quantitative Fecal Immunochemical Tests for Colorectal Cancer Screening. Gastroenterology. 2018;154(1):93-104.

2. Niedermaier T, Heisser T, Cardoso R, Hoffmeister M, Brenner H. Colonoscopy-Ascertained Prevalence of Advanced Neoplasia According to Fecal Hemoglobin Concentration in a Large Cohort of Fecal Immunochemical Test-Negative Screening Participants. Ann Intern Med. 2023;176(11):1569-71.

3. Brenner H, Altenhofen L, Stock C, Hoffmeister M. Prevention, early detection, and overdiagnosis of colorectal cancer within 10 years of screening colonoscopy in Germany. Clin Gastroenterol Hepatol. 2015;13(4):717-23.

4. Pox CP, Altenhofen L, Brenner H, Theilmeier A, Von Stillfried D, Schmiegel W. Efficacy of a nationwide screening colonoscopy program for colorectal cancer. Gastroenterology. 2012;142(7):1460-7.e2.

5. Chen H, Werner S, Brenner H. Fresh vs Frozen Samples and Ambient Temperature Have Little Effect on Detection of Colorectal Cancer or Adenomas by a Fecal Immunochemical Test in a Colorectal Cancer Screening Cohort in Germany. Clin Gastroenterol Hepatol. 2017;15(10):1547-56.e5.

6. Chung DC, Gray DM, Singh H, Issaka RB, Raymond VM, Eagle C, et al. A cell-free DNA blood-based test for colorectal cancer screening. New England Journal of Medicine. 2024;390(11):973-83.
